# Continuous hemodynamics monitoring during arteriovenous malformation microsurgical resection with laser speckle contrast imaging: case report

**DOI:** 10.3389/fsurg.2023.1285758

**Published:** 2023-12-15

**Authors:** Alexis Dimanche, David R. Miller, Johannes Goldberg, Andreas Raabe, Andrew K. Dunn, David Bervini

**Affiliations:** ^1^ Department of Biomedical Engineering, The University of Texas at Austin, Austin, TX, United States; ^2^ Dynamic Light Inc., Austin, TX, United States; ^3^ Department of Neurosurgery, Inselspital, Bern University Hospital, University of Bern, Bern, Switzerland

**Keywords:** arteriovenous malformation, blood flow imaging, case report, cerebral blood flow, continuous imaging, hemodynamics, laser speckle contrast imaging (LSCI)

## Abstract

AVM surgery is challenging due to progressive and often unforeseeable flow changes during its resection which involve both the AVM and the surrounding brain tissue. Hence, accurate monitoring of blood flow is crucial to minimize complications and improve outcomes. The following case report illustrates the usefulness of complimentary non-invasive tools that can provide real time blood flow assessment. We present a case demonstrating the application of laser speckle contrast imaging (LSCI) in evaluating vessel flow dynamics during AVM surgery. A 30-year-old female presented with sudden headaches, nausea, vomiting, and vertigo. Emergency imaging revealed a ruptured cerebellar AVM necessitating surgical intervention. LSCI was integrated into the surgical workflow, providing continuous visualization of relative cerebral blood flow (rCBF) of vessels surrounding the AVM. Before AVM resection, LSCI measurements revealed the arterialized vasculature supplying the AVM nidus; measurements after AVM resection showed significant hemodynamic changes including normal flow in the initially arterialized AVM draining veins and adjacent arterial branches. LSCI also detected blood flow alterations during temporary occlusion, enabling assessment of downstream vascular regions. In conclusion, we provide an example supporting the utility of LSCI for real-time hemodynamic monitoring during AVM resection surgery. LSCI offers non-invasive, continuous, and immediate blood flow information, complementing conventional imaging methods like indocyanine green angiography. Additionally, our findings suggest that LSCI has the potential to provide a non-invasive means of identifying the specific superficial vessel branches or cortical areas that receive blood supply from a particular vessel.

## 1. Introduction

Arteriovenous malformation (AVM) resection can be challenging and the understanding of vascular anatomy and hemodynamics during surgery is crucial to minimize morbidity. Indocyanine green angiography (ICGA) has become the gold-standard intraoperative blood flow monitoring tool in neurovascular surgeries, including AVM microsurgical resection (1–3). The use of intraoperative and semi-quantitative hemodynamic tools such as FLOW 800 (Carl Zeiss Meditec, Oberkochen, Germany), provide visualization of the vascular architecture and allows for accurate assessment of blood flow dynamics. Recently, laser speckle contrast imaging (LSCI) has been shown to be a promising tool for non-invasive, continuous, real-time, and full-field cerebral blood flow (CBF) monitoring (4, 5). LSCI utilizes the spatiotemporal fluctuations in the speckle pattern produced by the interaction of coherent laser light with moving red blood cells to calculate relative changes in blood flow, thus providing a noninvasive method for imaging microvascular blood flow in real-time. The technique provides semi-quantitative color-coded LSCI overlay videos throughout the surgery informing the surgeon about the CBF of the surgically exposed vasculature (6, 7). We present an AVM case where LSCI was used to continuously evaluate the evolving flow dynamics during the resection procedure. This case report has been reported in line with the CARE guidelines (8). The patient provided written informed consent the day prior to the surgery.

## 2. Case description

### 2.1. Patient information

**Figure 1:**
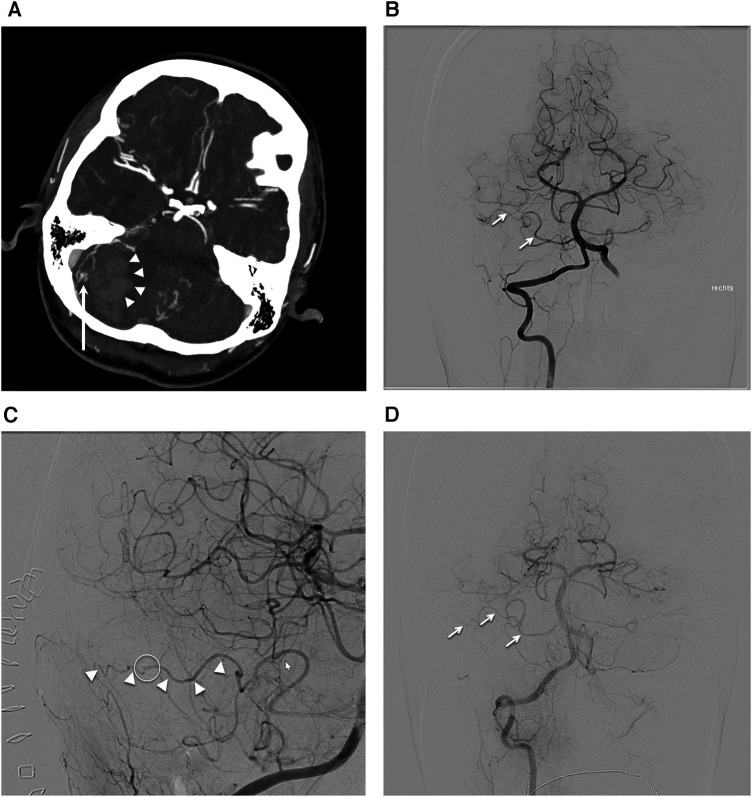
Case presentation. (**A**) Computed tomography angiography axial cut showing cerebral hematoma on the right-hand side (arrowheads) and associated arteriovenous malformation (AVM) nidus (arrow). (**B**) Anterior posterior projection of the preoperative digital subtraction angiography (DSA) after right vertebral artery injection displaying the AVM nidus being fed by the anterior inferior cerebellar artery (AICA) branches (arrows). (**C**) Oblique projection of the postoperative DSA after right vertebral artery injection showing the intact *en-passant* AICA (arrowheads) and the AVM clip on the previous AVM feeder (circle). (**D**) Anterior posterior projection of the DSA after right vertebral artery projection highlighting the intact *en-passant* AICA (arrows).

A 30-year-old female in her 30th week of pregnancy, presented at the emergency department with acute onsets of spontaneous headaches, nausea, vomiting and vertigo. Neurological examination showed signs of cerebellar dysfunction. A computed tomography angiography (CTA) was performed in the emergency setup revealing a cerebellar hematoma and associated small AVM nidus (Figure 1A). The patient was initially treated conservatively. Following an elective delivery, a diagnostic angiography was conducted to confirm the diagnosis of a Spetzler-Martin (SM) grade I, supplementary SM grade 3 right cerebellar AVM. Indication for surgical resection was given 6 months after initial hemorrhage and after confirming the diagnosis with a diagnostic subtraction angiography. The AVM was fed by *en-passant* branches of the anterior inferior cerebellar artery (AICA) (Figure 1B). The nidus was draining in a cerebellar cortical vein reaching the superior cerebellar vein and the transverse sinus on the right-hand side. A retrosigmoid craniotomy was performed and primary cortical vascular anatomy was exposed, using ICGA to differentiate the AICA branches from the AVM draining vein. The AVM nidus was progressively devascularized by disconnecting its AICA feeders but preserving the main *en-passant* AICA cortical branch (Figures 1C,D).

### 2.2. LSCI intraoperative imaging

The case was included in a single-center observational cohort study (ClinicalTrials.gov identifier: NCT0502840) which was approved by the local ethics committee in Bern, Switzerland under Project-ID: 2021-D0043. Throughout the treatment of the AVM, LSCI was used without informing surgical decisions. The LSCI instrumentation was adapted to the operating surgical microscope (Carl Zeiss Meditec, Oberkochen, Germany) as explained elsewhere (6). The intraoperative setup allowed continuous display of relative cerebral blood flow (rCBF) information on the operating room monitors.

### 2.3. Surgical intervention

**Figure 2:**
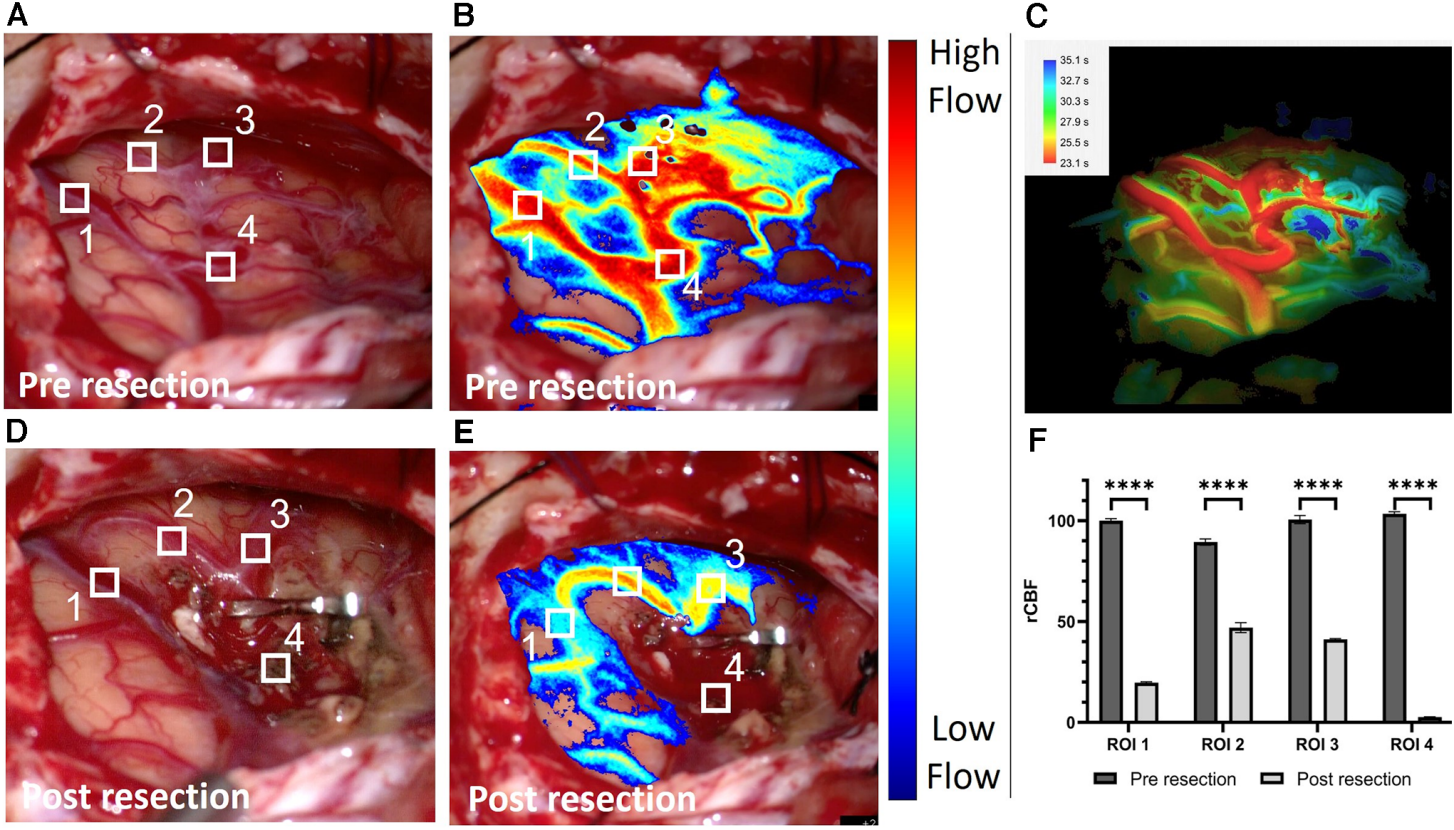
White light image of the surgical field pre resection (**A**) and associated laser speckle 233 contrast imaging (LSCI) overlay blood flow map (**B**). (**C**) Corresponding Flow 800 delay map (Carl 234 Zeiss Meditec, Oberkochen, Germany) computed <1 min after (**B**). White light image of the surgical field post resection (**D**) and associated LSCI overlay blood flow map (**E**). The clip is positioned on the arteriovenous malformation feeder leaving the en-passant branches (region of interest (ROI) 2 & 3) patent. The four ROIs are depicted and numbered on the white light and LSCI overlay images. (**F**) Grouped bar graph comparison between the normalized relative cerebral blood flow values in the vessel contained in the four ROIs pre and post resection. The inverse correlation time values computed from the speckle contrast are normalized to the pre resection data in ROI 1. A two-sample paired t-test was used to evaluate significance (*p* < 0.0001 for all four ROIs).

**Figure 3:**
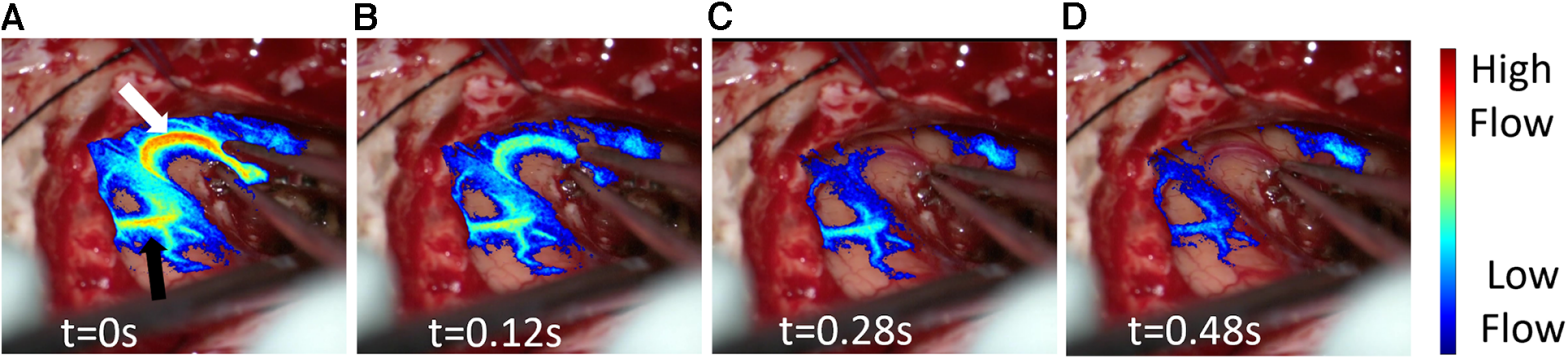
Post resection laser speckle contrast imaging (LSCI) overlay video demonstrating real time blood flow changes in response to the temporary occlusion on the artery. (**A**) LSCI overlay baseline frame (*t* = 0s) moments before complete artery occlusion. The white arrow highlights the occluded artery while the black arrow shows its downstream vascular region. (**B**–**D**) LSCI overlay frames at different timepoints during artery occlusion.

We utilized LSCI to measure rCBF before and after complete AVM resection as shown in Figure 2. The pre-resection baseline flow measurements (Figure 2B) provide similar and complementary information to ICGA and FLOW 800 (Figure 2C) highlighting the arterialized vasculature network feeding the AVM nidus. A large, arterialized vein (ROI 1 & 4) was draining the AVM nidus demonstrating high rCBF values similar to the arteries in ROI 2 and 3. The AVM resection resulted in significant hemodynamic changes across all ROIs. Notably, LSCI measured a decrease in flow (*p* < 0.001) in the initially arterialized vein (ROI 1) pre- and post-resection (Figure 2F) while capturing the absence of flow in the cauterized vessel (ROI 4). The *en-passant* arterial branches remained well perfused while exhibiting a decrease in flow compared to the pre-resection values. LSCI also accurately measured changes in blood flow due to temporary forceps occlusion with high temporal sensitivity (Figure 3) and informed about the downstream vascular regions supplied by the occluded artery. The post resection measurements were recorded right before fluorescein angiography evaluation. A post-operative angiography confirmed complete AVM resection and the post-operative course was uneventful, without new neurological deficits.

## 3. Discussion

In this report we further illustrate how LSCI can enhance the surgeon's assessment of CBF during AVM resection due to its continuous display of blood flow across the surgical field (4, 6, 9–11). The usefulness of LSCI in AVM embolization and resection has already been demonstrated by Tao et al. (12). However, due to the nature of the AVM surgery (hybrid) and the non-surgical microscope integrated LSCI instrumentation, LSCI could not be used to monitor CBF throughout the entire surgery and offer real-time visualization of CBF changes. We focus on analyzing the evolving flow dynamics in an arterialized vein during AVM resection and provide a video demonstrating continuous monitoring of flow changes during temporary vessel occlusion. This expands on the findings in Miller et al. (6) which are limited to a pre-and-post-resection rCBF comparison in a single ROI and did not capture the real-time hemodynamic transformations occurring during the resection process. Continuous monitoring with LSCI enables gradual assessment of the decrease of blood flow in the vein over time while monitoring the patency of other regions of interest. We measured a significant decrease in flow in two *en-passant* artery branches neighboring the nidus (ROI 2 & 3). This is possibly related to a reduction of main flow and “steal” effect at the time of complete AVM devascularization.

In addition to rCBF comparisons at different time points in the surgery, LSCI revealed instant blood flow changes in response to a temporary occlusion (Figure 3, Supplementary Video S1). The blood flow changes in the occluded *en-passant* arterial branch (Figure 3A, white arrow) were measured with excellent temporal and spatial resolution. As depicted in Figure 3, LSCI showed that the vessel (Figure 3A, black arrow) is situated downstream of the occluded artery as it also experienced a significant decrease in flow during temporary occlusion. Such a feature may enable a non-invasive way to check which superficial vessel branches or areas of the cortex are fed by a certain vessel. For example, intraoperatively, there remains uncertainty in identifying *en-passant* vessels and identifying their downstream behavior and location.

Although not highlighted in this report, LSCI is also a promising tool to monitor cortical perfusion throughout the surgery and inform about significant and unexpected changes (7). Continuous cortical perfusion monitoring may benefit AVM surgery in the early detection of downstream cortical hypoperfusion while occluding *en-passant* arteries not directly involved in AVM nidus perfusion.

The main limitation of LSCI in AVM surgery is its limited penetration depth (<1 mm) which only offers superficial blood flow information of surgically visible vasculature (5, 13). Hence, LSCI shares this shortcoming with fluorescence angiography (FA) as it is unable to evaluate residual flow in deep parts of the nidus. This confirmation can only be obtained through digital subtraction angiography, the standard but more expensive modality.

FA and LSCI complement each other, with the latter offering additional information throughout surgery to guide procedures and inform surgeons about hemodynamic changes. However, for critical tasks like determining flow direction and distinguishing between arteries and veins, the bolus injection and fluorescence delay of FA are indispensable, providing information currently unavailable with LSCI. Additionally, FA plays a crucial role in determining whether a vessel is arterialized and to confirm total resection (2, 3).

More thorough clinical trials are necessary to evaluate the accuracy and usefulness of LSCI in AVM surgery. This report provides an additional, compelling illustration of the utility of continuous blood low monitoring with LSCI during AVM surgery.

## 4. Conclusion

The present example is another illustration of the usefulness of LSCI for measuring the progressive devascularization of AVM and reduction of the arterialized blood flow in the AVM draining veins. Specifically, we highlight its role in guiding the surgeon to better understand the complex hemodynamics involved in AVM surgery. The continuous, full-field, and non-invasive blood flow monitoring technique was integrated into the surgical workflow and measured relevant CBF changes.

## Data Availability

The original contributions presented in the study are included in the article/**Supplementary Material**, further inquiries can be directed to the corresponding author.

## Supplementary material

Supplementary Video S1 Post resection laser speckle contrast imaging (LSCI) overlay video demonstrating real time blood flow changes in response to the temporary occlusion on the artery.

## References

[1] Faber F Thon N Fesl G Rachinger W Guckler R Tonn J-C Enhanced analysis of intracerebral arterioveneous malformations by the intraoperative use of analytical indocyanine green videoangiography: technical note. Acta Neurochir (Wien). (2011) 153:2181–7. 10.1007/s00701-011-1141-z 21894496

[2] Fukuda K Kataoka H Nakajima N Masuoka J Satow T Iihara K. Efficacy of FLOW 800 with indocyanine green videoangiography for the quantitative assessment of flow dynamics in cerebral arteriovenous malformation surgery. World Neurosurg. (2015) 83:203–10. 10.1016/j.wneu.2014.07.012 25045789

[3] Acerbi F Vetrano IG Sattin T Falco J de Laurentis C Zattra CM Use of ICG videoangiography and FLOW 800 analysis to identify the patient-specific venous circulation and predict the effect of venous sacrifice: a retrospective study of 172 patients. Neurosurg Focus. (2018) 45:E7. 10.3171/2018.4.FOCUS18120 29961380

[4] Boas DA Dunn AK. Laser speckle contrast imaging in biomedical optics. J Biomed Opt. (2010) 15:011109. 10.1117/1.3285504 20210435 PMC2816990

[5] Senarathna J Rege A Li N Thakor NV. Laser speckle contrast imaging: theory, instrumentation and applications. IEEE Rev Biomed Eng. (2013) 6:99–110. 10.1109/RBME.2013.2243140 23372086

[6] Miller DR Ashour R Sullender CT Dunn AK. Continuous blood flow visualization with laser speckle contrast imaging during neurovascular surgery. Neurophotonics. (2022) 9:021908. 10.1117/1.nph.9.2.021908 35265733 PMC8900813

[7] Hecht N Woitzik J König S Horn P Vajkoczy P. Laser speckle imaging allows real-time intraoperative blood flow assessment during neurosurgical procedures. J Cereb Blood Flow Metab. (2013) 33:1000–7. 10.1038/jcbfm.2013.42 23512134 PMC3705427

[8] Gagnier JJ Kienle G Altman DG Moher D Sox H Riley D. The CARE guidelines: consensus-based clinical case reporting guideline development. Glob Adv Health Med. (2013) 2:38–43. 10.7453/gahmj.2013.008 24416692 PMC3833570

[9] Parthasarathy AB Weber EL Richards LM Fox DJ Dunn AK. Laser speckle contrast imaging of cerebral blood flow in humans during neurosurgery: a pilot clinical study. J Biomed Opt. (2010) 15:066030. 10.1117/1.3526368 21198204 PMC9113397

[10] Hecht N Woitzik J Dreier JP Vajkoczy P. Intraoperative monitoring of cerebral blood flow by laser speckle contrast analysis. Neurosurg Focus. (2009) 27:E11. 10.3171/2009.8.FOCUS09148 19795950

[11] Towle EL Richards LM Kazmi SMS Fox DJ Dunn AK. Comparison of indocyanine green angiography and laser speckle contrast imaging for the assessment of vasculature perfusion. Neurosurgery. (2012) 71:1023–30. 10.1227/NEU.0b013e31826adf88 22843129 PMC3718844

[12] Tao S Zhang T Zhou K Liu X Feng Y Zhao W Intraoperative monitoring cerebral blood flow during the treatment of brain arteriovenous malformations in hybrid operating room by laser speckle contrast imaging. Front Surg. (2022) 9:855397. 10.3389/fsurg.2022.855397 35599788 PMC9120635

[13] Davis MA Kazmi SMS Dunn AK. Imaging depth and multiple scattering in laser speckle contrast imaging. J Biomed Opt. (2014) 19:086001. 10.1117/1.jbo.19.8.086001 25089945 PMC4119427

